# Emollient enhancement of the skin barrier from birth offers effective atopic dermatitis prevention

**DOI:** 10.1016/j.jaci.2014.08.005

**Published:** 2014-10

**Authors:** Eric L. Simpson, Joanne R. Chalmers, Jon M. Hanifin, Kim S. Thomas, Michael J. Cork, W.H. Irwin McLean, Sara J. Brown, Zunqiu Chen, Yiyi Chen, Hywel C. Williams

**Affiliations:** a Department of Dermatology, Oregon Health & Science University, Portland, Ore; e Oregon Clinical & Translational Research Institute, Oregon Health & Science University, Portland, Ore; f Public Health & Preventive Medicine, Oregon Health & Science University, Portland, Ore; b Centre of Evidence Based Dermatology, University of Nottingham, Nottingham, United Kingdom; c Dermatology Research, Department of Infection and Immunity, University of Sheffield, Sheffield, United Kingdom; d Dermatology & Genetic Medicine, University of Dundee, Dundee, United Kingdom

**Keywords:** Atopic dermatitis, eczema, skin barrier, prevention, emollients

## Abstract

**Background:**

Atopic dermatitis (atopic eczema) is a chronic inflammatory skin disease that has reached epidemic proportions in children worldwide and is increasing in prevalence. Because of the significant socioeconomic effect of atopic dermatitis and its effect on the quality of life of children and families, there have been decades of research focused on disease prevention, with limited success. Recent advances in cutaneous biology suggest skin barrier defects might be key initiators of atopic dermatitis and possibly allergic sensitization.

**Objective:**

Our objective was to test whether skin barrier enhancement from birth represents a feasible strategy for reducing the incidence of atopic dermatitis in high-risk neonates.

**Methods:**

We performed a randomized controlled trial in the United States and United Kingdom of 124 neonates at high risk for atopic dermatitis. Parents in the intervention arm were instructed to apply full-body emollient therapy at least once per day starting within 3 weeks of birth. Parents in the control arm were asked to use no emollients. The primary feasibility outcome was the percentage of families willing to be randomized. The primary clinical outcome was the cumulative incidence of atopic dermatitis at 6 months, as assessed by a trained investigator.

**Results:**

Forty-two percent of eligible families agreed to be randomized into the trial. All participating families in the intervention arm found the intervention acceptable. A statistically significant protective effect was found with the use of daily emollient on the cumulative incidence of atopic dermatitis with a relative risk reduction of 50% (relative risk, 0.50; 95% CI, 0.28-0.9; *P* = .017). There were no emollient-related adverse events and no differences in adverse events between groups.

**Conclusion:**

The results of this trial demonstrate that emollient therapy from birth represents a feasible, safe, and effective approach for atopic dermatitis prevention. If confirmed in larger trials, emollient therapy from birth would be a simple and low-cost intervention that could reduce the global burden of allergic diseases.

Atopic dermatitis (atopic eczema) is a chronic inflammatory skin disease that has reached epidemic proportions in children worldwide and is increasing in prevalence.^1^, ^2^ Children with atopic dermatitis experience intractable itch along with inflamed, cracked, and often infected skin lesions. The onset of atopic dermatitis in childhood often heralds the development of subsequent allergic disorders, such as food allergy, asthma, and allergic rhinitis (the atopic march), as well as neurodevelopmental disorders.^3^, ^4^ Development of an effective prevention strategy for atopic dermatitis and associated allergic disease would represent a major public health breakthrough.

Atopic dermatitis has been historically classified as an allergic disease, given its association with IgE-mediated diseases, such as food allergy. Prevention trials to date have primarily focused on allergen avoidance. Unfortunately, the results of these studies have been largely disappointing or inconsistent, and no single accepted prevention strategy has emerged.^5^

Recent advances in cutaneous biology suggest epidermal defects might be a key initiator of atopic dermatitis and possibly allergic sensitization.^6^, ^7^, ^8^ Skin barrier dysfunction is now recognized as central to the initiation and progression of atopic dermatitis. These new findings create an opportunity for the development of novel prevention strategies focusing on the skin barrier. We hypothesize that enhancement of a defective skin barrier early in life might prevent or delay the onset of atopic dermatitis.

Emollients provide a safe and effective method of skin barrier enhancement because they provide the skin with a source of exogenous lipids, improving its barrier properties.^9^, ^10^, ^11^ The results of a previous case-control study and open-label trial suggest the use of bland emollients from birth might protect against the onset of skin inflammation in neonates.^12^, ^13^ The objective for this study was to test the hypothesis that emollient therapy from birth represents a safe, feasible, and efficacious approach to the prevention of atopic dermatitis (Fig 1).

**Fig 1:**
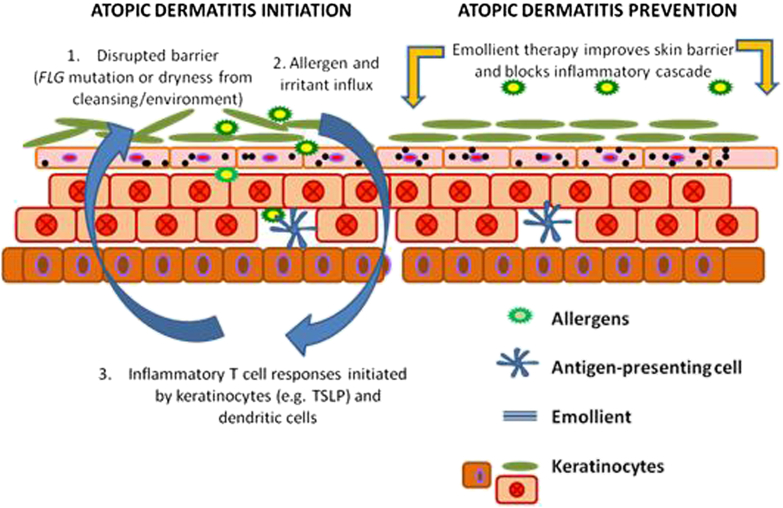
Skin barrier protection might prevent atopic dermatitis development. *FLG*, Filaggrin.

## Methods

### Study design

This was a multicenter, multinational, 2-arm parallel-group, assessor-blind, randomized (1:1) controlled pilot trial of 6 months' duration. The intervention started within 3 weeks of birth.

### Participants

Infants at high risk of eczema, which was defined as having a parent or full sibling who has (or had) physician-diagnosed atopic dermatitis, asthma, or allergic rhinitis, were included. The strongest and most established risk factor for atopic dermatitis is a family history of atopic disease.^14^, ^15^ Thus to qualify for this study, neonates had to have had 1 first-degree relative with a history of allergic rhinitis, asthma, or atopic dermatitis. Between 25% and 40% of children with a family history of atopic disease have atopic dermatitis in the first year of life, with some reports putting the risk at greater than 60%.^16^ Infants needed to be in overall good health, and the mother needed to be at least 16 years of age at delivery and capable of providing informed consent. If mothers had taken *Lactobacillus rhamnosus* supplements during pregnancy, their infants were excluded. Infants were excluded if they were born before 37 weeks' gestation or if they had a major congenital anomaly, hydrops fetalis, an immunodeficiency syndrome, a severe genetic skin disorder, or a serious skin condition that would make the use of emollients inadvisable.

### Intervention

Parents in the intervention group were offered a choice of 3 emollients of different viscosities (an oil, a cream/gel, or an ointment) that had been selected based on previous data regarding their safety, tolerability, or barrier-protective qualities.^17^, ^18^, ^19^, ^20^, ^21^, ^22^ In the United Kingdom emollient choices were sunflower seed oil (William Hodgson and Co, Congleton, United Kingdom), Doublebase Gel (Dermal Laboratories, Hitchin, United Kingdom), and liquid paraffin 50% in white soft paraffin. In the United States parents were offered the same sunflower seed oil as used in the United Kingdom, Cetaphil Cream (Galderma Laboratories, Fort Worth, Tex), or Aquaphor Healing Ointment (Beiersdorf, Chester, Ohio). We used sunflower seed oil with a high ratio of linoleic/oleic acid to optimize the positive skin barrier effects.^23^ None of the emollients offered contain sodium lauryl sulfate because this emulsifier has been shown to adversely affect the skin barrier.^24^ Parents were asked to apply the emollient to the baby's entire body surface, with the exception of the scalp, starting as soon as possible after birth (within a maximum of 3 weeks) and continuing until the infant was 6 months of age.

Both the intervention and control groups were given an infant skin care advice booklet, which reflected current guidelines.^25^ Parents are advised (1) to avoid soap and bubble bath; (2) use a mild, fragrance-free synthetic cleanser designed specifically for babies; (3) avoid bath oils and additives; (4) use a mild, fragrance-free shampoo designed specifically for babies and avoid washing the suds over the baby's body; and (5) avoid using baby wipes, where possible.

### Outcomes

The primary purpose of this trial was to determine the feasibility of this approach for atopic dermatitis prevention in preparation for larger trials. Thus the primary outcome for this pilot study was the proportion of eligible families who were willing to be randomized.

Secondary outcomes were as follows:

• proportion of families eligible for the trial;
• proportion of families accepting the initial invitation to participate;
• percentage of early withdrawals;
• proportion of families who found the intervention acceptable;
• reported adherence with intervention;
• amount of contamination in the control group;
• age of onset of eczema and proportion of transient cases;
• incidence of emollient-related adverse events;
• success of blinding of the assessor to allocation status; and
• cumulative incidence of eczema at 6 months, as determined by an investigator.

Filaggrin mutation testing was performed in the McLean laboratory (Dundee, United Kingdom), evaluating the 4 loss-of-function mutations (R501X, 2282del4, S3247X, and R2447X) that are most prevalent in populations of white European ancestry by using TaqMan allelic discrimination (Thermo Fisher Scientific, Waltham, Mass), as described previously.^26^

### Recruitment and setting

Recruitment took place in the United Kingdom and the United States between May 2010 and May 2011. In the United Kingdom research nurses were based in 3 acute National Health Service hospital trusts (Nottingham University Hospitals, Derby Hospitals, and United Lincolnshire Hospitals) and 1 general practice surgery (the Surgery@Wheatbridge, Chesterfield). In the United States the study recruited in 1 hospital, Oregon Health & Science University Hospital and Clinics (Portland, Oregon).

### Visit schedule and randomization

Participation in the trial was for 6 months' duration. Methods of identifying suitable families differed between the United Kingdom and the United States. In the United Kingdom families were usually identified and screened during pregnancy by means of advertisement. After the family had made contact with the study team and initial eligibility checks had been carried out by the coordinating center, the research nurse carried out the screening and consent visit, usually at the family home. The baseline visit, including randomization, then took place within 3 weeks of delivery, usually as a home visit. In the United States families were identified by study coordinators visiting the postnatal wards each day and approaching parents about the study directly. After giving parents time to consider the study, the study coordinators returned to the family to obtain written consent and randomize the subjects.

Infants were randomized at a 1:1 ratio using random block sizes to either the intervention or control group with a central, Web-based, computer-generated, Internet randomization service provided by the Nottingham Clinical Trials Unit. The allocation list was held by the Nottingham Clinical Trials Unit and concealed from trial investigators and other trial staff. Allocation was only released to the research nurse by telephone once eligible participants' details were irrevocably entered into the online database by the coordinating center staff. Randomization was stratified by the recruiting research nurse. In the case of multiple births, the firstborn was the index child.

The research nurse contacted parents by telephone at 10 days and 6 weeks, with a face-to-face visit at 12 weeks (usually at home in the United Kingdom and as a clinic visit in the United States). This was then followed by a further telephone call at 18 weeks, and the final contact was a clinic visit at 24 weeks for an assessment by the dermatologist or dermatology specialist nurse, who conducted a blinded assessment of the skin. In addition to these scheduled contact points, parents were encouraged to contact the research nurse if they had any concerns about the child's skin. If parents reported symptoms of eczema, then an unscheduled visit to the hospital to see the dermatologist was arranged so that the presence of eczema could be confirmed.

### Blinding

It was not possible to blind parents in a trial of daily emollient application. An independent outcome assessor who was blinded to treatment allocation performed the skin examinations and diagnosis of eczema. This was usually a general practitioner, dermatologist, or dermatology nurse specialist. The statistician was blinded to treatment group until the analysis was complete.

### Approvals

The study was given ethical approval by the Nottingham Research Ethics Committee 1 in the United Kingdom (reference 09/H0407/43) and the Oregon Health & Science University Institutional Review Board in the United States and approved by all participating institutions. The trial was registered at Current Controlled Trials (ISRCTN84854178).

### Sample size

This was a pilot study and therefore not powered to establish the effectiveness of the intervention. The sample size was dictated by the time and resources available and was based on the primary outcome of willingness to randomize. Use of 100 families was calculated to provide an estimate within 10 percentage points for a 95% CI of the proportion willing to be randomized, assuming between 40% and 60% were willing to be randomized.

### Data analysis

The main clinical end point was the week 24 cumulative incidence of physician-diagnosed eczema. Infants were classified as having eczema if either the investigator or another medically qualified person (eg, a general practitioner) judged that the infant had eczema on examination at any point during the 24-week intervention period. The primary clinical end point analysis was conducted on an intent-to-treat basis using a complete case approach. Multiple sensitivity analyses were performed for the primary clinical end point. Missing data were imputed for infants with missing week 24 skin examination data because they were either lost to follow-up or withdrew during the 24-week intervention period (for reasons other than developed eczema). The following imputations were performed.

#### Multiple imputation

SAS procedure PROC MI was used to impute the missing data by using the Markov Chain Monte Carlo method, assuming the data are missing at random. By using variables from a logistic regression model, multiple imputation was performed to replace each missing values with 4 imputed values. SAS procedure PROC MIANALYSIS was used to combine the results from 5 imputed data sets for final inference results. Baseline variables used for the multiple imputation model were as follows: intervention group, study center, paternal asthma, maternal asthma, and paternal eczema. This combination of variables was found to best predict the outcome of eczema by using logistic regression with the smallest Akaike information criterion.

#### Imputated data sets

*Eczema imputation* was defined as all those with missing data assumed to have eczema. *No eczema imputation* was defined as all those with missing data assumed to not have eczema. *Worst-case imputation* was defined as all those with missing data in the emollient arm assumed to have eczema and all those in the control arm assumed to not have eczema (worst case). *Best-case imputation* was defined as all those with missing data in the control arm assumed to have eczema and all those in the emollient arm assumed to not have eczema (best case).

## Results

### Feasibility end points

**Fig 2:**
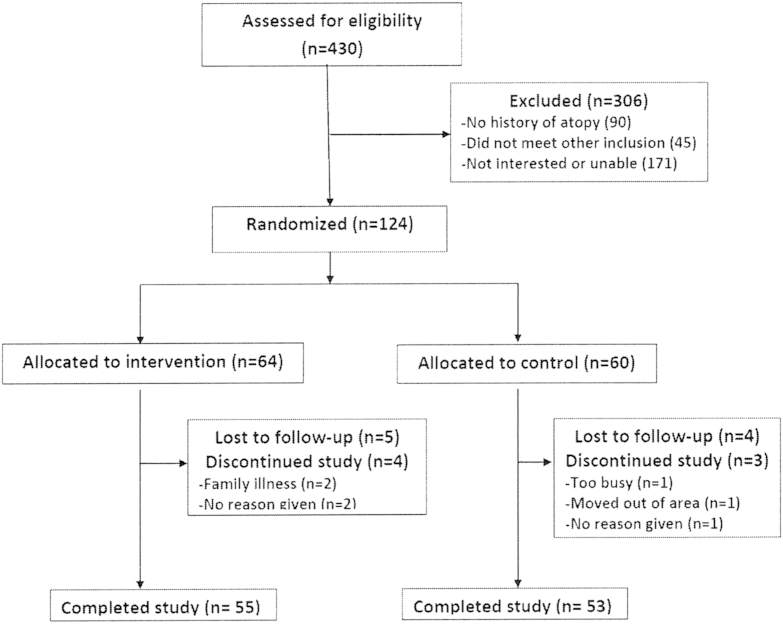
CONSORT flow diagram.

A total of 430 families were identified, of which 135 (31%) were not eligible. Of the 295 eligible families, 124 (42%) accepted the initial invitation to participate and were randomized (see the CONSORT flow diagram, Fig 2). The planned sample size was exceeded to allow all sites to enroll an adequate sample. Baseline characteristics were similar between treatment groups (Table I). The number of babies with a loss-of-function mutation in the skin barrier gene filaggrin, the strongest known genetic predictor of atopic dermatitis,^27^ was also similar between the 2 groups. By 6 months, 9 participants in the intervention arm and 7 in the control arm were lost to follow-up or withdrew in the intervention arm (12.9% attrition). All parents reported they found the emollient “acceptable,” and none of the families withdrew because of the emollient.

**Table I:** Baseline characteristics

Characteristic	Total no.	Control, no. (%), n = 60	Emollient, no. (%), n = 64
Mother with eczema	124	27 (45.0)	23 (35.9)
Mother with asthma	124	23 (38.3)	18 (28.1)
Father with eczema	124	13 (21.7)	16 (25.0)
Father with asthma	124	10 (16.7)	14 (21.9)
At least 1 parent with eczema	124	30 (50.0)	33 (51.6)
Both parents with eczema	124	5 (8.3)	3 (4.7)
Cesarean delivery	124	17 (28.3)	16 (25.0)
Any *FLG* mutation	95	8 (17.8)	13 (26.0)
*FLG* heterozygous	95	6 (13.3)	13 (26.0)
*FLG* homozygous/compound heterozygous	95	2 (4.4)	0 (0.0)

*FLG*, Filaggrin.

In the intervention group the cream/gel formulation was the preferred emollient (67.2%), followed by oil (23.4%) and then ointment (9.4%). Approximately 85% of parents in the intervention group reported using emollients at least 5 days per week in the intervention group at 6 months (Table II). Eight (13.3%) parents in the control group reported using emollients in a way that mirrored the intervention (ie, regular generalized application of emollient for reasons other than the treatment of cradle cap, nappy rash, or eczema). These participants remained as control subjects in the analyses based on the intent-to-treat principle. The blinding was not maintained for 5 (4%) of 124 skin examinations, and this was usually because the infant had already been treated by the assessor.

**Table II:** Emollient group adherence

Emollient use	Week 6∗	Week 12	Week 18∗	Week 24
None	0	0	1 (1.8%)	0
1-2 d/wk	1 (1.9%)	0	1 (1.8%)	3 (5.6%)
3-4 d/wk	4 (7.4%)	5 (9.1%)	5 (8.8%)	5 (9.3%)
5-6 d/wk	7 (13.0%)	8 (14.6%)	7 (12.3%)	2 (3.7%)
Everyday	42 (77.8%)	42 (76.4%)	43 (75.4%)	44 (81.5%)
Total	54	55	57	54

∗ Telephone visit.

### Clinical end points

Daily emollient use significantly reduced the cumulative incidence of atopic dermatitis at 6 months (43% in the control group vs 22% in the emollient group). This corresponds to a relative risk reduction of 50% (relative risk, 0.50; 95% CI, 0.28-0.90; *P* = .017). A sensitivity analysis using a multiple imputation approach for missing data from the 16 participants who withdrew or were lost to follow-up showed a similar statistically significant protective effect of emollient therapy as the complete case results (data not shown). Further sensitivity analyses were performed by imputing missing data, assuming those with missing data either all had eczema or all did not have eczema, and found similar significant results in 3 of the 4 scenarios (Table III). We were unable to assess the age of onset of eczema (and thus assess whether cases were transient) with any precision because of the low numbers of subjects who presented with eczema symptoms before the 6-month visit (data not shown). Three superficial cutaneous infections occurred in each group, all of which were considered mild in nature. There were no reports of irritant or allergic contact dermatitis.

**Table III:** Additional sensitivity analyses

Case	Total no.	Had AD (control group)	Had AD (emollient group)	RR (95% CI)	*P* value
Complete (no imputation)	108	23/53 (43.4%)	12/55 (21.8%)	0.50 (0.28-0.90)	.017
Missing (assumed to develop AD)	124	30/60 (50.0%)	21/64 (32.8%)	0.66 (0.43-1.01)	.03
Missing (assumed not to develop AD)	124	23/60 (38.3%)	12/64 (18.8%)	0.49 (0.27-0.89)	.02
Worst-case scenario∗	124	23/60 (38.3%)	21/64 (32.8%)	1.0 (0.61-1.62)	.99
Best-case scenario†	124	30/60 (50.0%)	12/64 (18.8%)	0.38 (0.2-0.66)	<.001

*AD*, Atopic dermatitis; *RR*, relative risk.

∗ Missing in emollient group assumed to have AD and in control group assumed not to have AD.

† Missing in emollient group assumed not to have AD and in control group assumed to have AD.

## Discussion

This study provides the first randomized controlled trial evidence that daily full-body emollient therapy from birth can prevent atopic dermatitis. In our study, parents adhered adequately to the simple intervention, and no significant adverse events occurred. The morbidity of atopic dermatitis and the increasing prevalence and potential toxicity of current immunosuppressant therapies make disease prevention an important goal. Atopic dermatitis prevention was listed as an “urgent call for research” in the United Kingdom Health Technology Assessment Systematic Review of Atopic Dermatitis Therapy published more than decade ago.^28^ However, despite the publication of more than 100 randomized clinical trials on the prevention of atopic dermatitis, the majority of which have evaluated allergen avoidance preventive strategies, no strategy has been generally accepted as effective.^5^

Although not previously studied for primary prevention, emollient therapy plays an integral role in the management of established atopic dermatitis.^29^ The exact mechanisms through which emollients exert their positive effects are not completely understood. We propose that emollients correct subclinical skin barrier dysfunction and early inflammation in predisposed infants before atopic dermatitis development by improving skin hydration and reducing skin permeability. This skin barrier enhancement prevents skin dryness and cracking, as well as inhibiting irritant and allergen penetration into the epidermis, which are potential initiators of skin inflammation.

Future studies should address the potential for skin barrier protection to reduce IgE sensitization. Both human and mouse studies suggest that the skin barrier might be a site for IgE sensitization.^30^, ^31^, ^32^ If so, this approach might also represent a novel allergic asthma and food allergy prevention strategy. More data regarding the optimum emollients for atopic dermatitis prevention are also needed. Emollients should improve skin barrier function, be free of irritants and potential allergens, and be low cost and easy to use so that the intervention can be used worldwide. It is unclear whether formulations that contain special additives, such as ceramides, improve the skin barrier or provide better clinical outcomes than simple petrolatum-based emollients.

The strengths of our study include the randomized controlled trial design, blinded outcome assessment, and potential external validity of studying the intervention in 2 countries and several centers. The major limitation of this study was the short follow-up time and small number of participants. Because it is possible that the use of emollients in our pilot study simply masked very mild eczema by exerting a weak anti-inflammatory effect, long-term follow-up beyond the intervention is crucial for any future randomized trials, and such a study is underway in the United Kingdom.

**Clinical implications**

Emollient therapy from birth represents a novel approach to atopic dermatitis primary prevention. We anticipate these data will encourage larger trials of this approach in various populations.

## References

[1] Odhiambo J.A., Williams H.C., Clayton T.O., Robertson C.F., Asher M.I. (2009). ISAAC Phase Three Study Group. Global variations in prevalence of eczema symptoms in children from ISAAC phase three. J Allergy Clin Immunol.

[2] Williams H., Stewart A., von Mutius E., Cookson W., Anderson H.R. (2008). International Study of Asthma and Allergies in Childhood (ISAAC) Phase One and Three Study Groups. Is eczema really on the increase worldwide?. J Allergy Clin Immunol.

[3] Yaghmaie P., Koudelka C.W., Simpson E.L. (2013). Mental health comorbidity in patients with atopic dermatitis. J Allergy Clin Immunol.

[4] Spergel J.M. (2010). From atopic dermatitis to asthma: the atopic march. Ann Allergy Asthma Immunol.

[5] Foisy M., Boyle R.J., Chalmers J.R., Simpson E.L., Williams H.C. (2011). Overview of reviews the prevention of eczema in infants and children: an overview of Cochrane and non-Cochrane reviews. Evid Based Child Health.

[6] Boguniewicz M., Leung D.Y. (2011). Atopic dermatitis: a disease of altered skin barrier and immune dysregulation. Immunol Rev.

[7] Leung D.Y. (2013). New insights into atopic dermatitis: role of skin barrier and immune dysregulation. Allergol Int.

[8] Brown S.J., McLean W.H. (2012). One remarkable molecule: Filaggrin. J Invest Dermatol.

[9] Loden M. (2003). Role of topical emollients and moisturizers in the treatment of dry skin barrier disorders. Am J Clin Dermatol.

[10] Wigger-Alberti W., Elsner P. (1997). Petrolatum prevents irritation in a human cumulative exposure model in vivo. Dermatology.

[11] Ghali F.E. (2005). Improved clinical outcomes with moisturization in dermatologic disease. Cutis.

[12] Simpson E.L., Berry T.M., Brown P.A., Hanifin J.M. (2010). A pilot study of emollient therapy for the primary prevention of atopic dermatitis. J Am Acad Dermatol.

[13] Macharia W.M., Anabwani G.M., Owili D.M. (1991). Effects of skin contactants on evolution of atopic dermatitis in children: a case control study. Trop Doct.

[14] Apfelbacher C.J., Diepgen T.L., Schmitt J. (2011). Determinants of eczema: population-based cross-sectional study in Germany. Allergy.

[15] Eichenfield L.F., Tom W.L., Chamlin S.L., Feldman S.R., Hanifin J.M., Simpson E.L. (2014). Guidelines of care for the management of atopic dermatitis: Section 1. diagnosis and assessment of atopic dermatitis. J Am Acad Dermatol.

[16] Simpson E.L., Keck L.E., Chalmers J.R., Williams H.C. (2012). How should an incident case of atopic dermatitis be defined? A systematic review of primary prevention studies. J Allergy Clin Immunol.

[17] Darmstadt G.L., Mao-Qiang M., Chi E., Saha S.K., Ziboh V.A., Black R.E. (2002). Impact of topical oils on the skin barrier: Possible implications for neonatal health in developing countries. Acta Paediatr.

[18] Darmstadt G.L., Badrawi N., Law P.A., Ahmed S., Bashir M., Iskander I. (2004). Topically applied sunflower seed oil prevents invasive bacterial infections in preterm infants in Egypt: a randomized, controlled clinical trial. Pediatr Infect Dis J.

[19] Edwards W.H., Conner J.M., Soll R.F. (2004). Vermont Oxford Network Neonatal Skin Care Study Group. The effect of prophylactic ointment therapy on nosocomial sepsis rates and skin integrity in infants with birth weights of 501 to 1000 g. Pediatrics.

[20] Ghadially R., Halkier-Sorensen L., Elias P.M. (1992). Effects of petrolatum on stratum corneum structure and function. J Am Acad Dermatol.

[21] Laquieze S., Czernielewski J., Baltas E. (2007). Beneficial use of cetaphil moisturizing cream as part of a daily skin care regimen for individuals with rosacea. J Dermatolog Treat.

[22] Lane A.T., Drost S.S. (1993). Effects of repeated application of emollient cream to premature neonates' skin. Pediatrics.

[23] Danby S.G., AlEnezi T., Sultan A., Lavender T., Chittock J., Brown K. (2013). Effect of olive and sunflower seed oil on the adult skin barrier: implications for neonatal skin care. Pediatr Dermatol.

[24] Danby S.G., Al-Enezi T., Sultan A., Chittock J., Kennedy K., Cork M.J. (2011). The effect of aqueous cream BP on the skin barrier in volunteers with a previous history of atopic dermatitis. Br J Dermatol.

[25] Blume-Peytavi U., Cork M., Faergemann J., Szczapa J., Vanaclocha F., Gelmetti C. (2009). Bathing and cleansing in newborns from day 1 to first year of life: recommendations from a European round table meeting. J Eur Acad Dermatol Venereol.

[26] Sandilands A., Terron-Kwiatkowski A., Hull P.R., O'Regan G.M., Clayton T.H., Watson R.M. (2007). Comprehensive analysis of the gene encoding filaggrin uncovers prevalent and rare mutations in ichthyosis vulgaris and atopic eczema. Nat Genet.

[27] McAleer M.A., Irvine A.D. (2013). The multifunctional role of filaggrin in allergic skin disease. J Allergy Clin Immunol.

[28] Hoare C., Li Wan Po A., Williams H. (2000). Systematic review of treatments for atopic eczema. Health Technol Assess.

[29] Szczepanowska J., Reich A., Szepietowski J.C. (2008). Emollients improve treatment results with topical corticosteroids in childhood atopic dermatitis: a randomized comparative study. Pediatr Allergy Immunol.

[30] Fallon P.G., Sasaki T., Sandilands A., Campbell L.E., Saunders S.P., Mangan N.E. (2009). A homozygous frameshift mutation in the mouse flg gene facilitates enhanced percutaneous allergen priming. Nat Genet.

[31] Herrick C.A., MacLeod H., Glusac E., Tigelaar R.E., Bottomly K. (2000). Th2 responses induced by epicutaneous or inhalational protein exposure are differentially dependent on IL-4. J Clin Invest.

[32] Spergel J.M., Mizoguchi E., Brewer J.P., Martin T.R., Bhan A.K., Geha R.S. (1998). Epicutaneous sensitization with protein antigen induces localized allergic dermatitis and hyperresponsiveness to methacholine after single exposure to aerosolized antigen in mice. J Clin Invest.

